# Pre-operative and intra-operative detection of axillary lymph node metastases in 108 patients with invasive lobular breast cancer undergoing mastectomy

**DOI:** 10.1186/s12885-018-4062-x

**Published:** 2018-02-05

**Authors:** Jerica Novak, Nikola Besic, Radan Dzodic, Barbara Gazic, Andrej Vogrin

**Affiliations:** 1 Department of Surgical Oncology, Institute of Oncology Ljubljana, Zaloska cesta 2, 1000, Ljubljana, Slovenia; 2 Department of Surgical Oncology, Institute of Oncology and Radiology of Serbia, Pasterova 14, Belgrade, Serbia; 3 Department of Pathology, Institute of Oncology Ljubljana, Zaloska cesta 2, 1000 Ljubljana, Slovenia; 4 Department of Radiology, Institute of Oncology Ljubljana, Zaloska cesta 2, 1000 Ljubljana, Slovenia

**Keywords:** Invasive lobular carcinoma, Axillary lymph node metastasis, Axillary ultrasound, Ultrasound-guided fine-needle aspiration biopsy, Intra-operative imprint cytology

## Abstract

**Background:**

Despite the recent changes in the treatment of the axilla in selected breast cancer patient, positive sentinel lymph node (SLN) in patients undergoing mastectomy still necessitates axillary lymph node dissection (ALND). In invasive lobular carcinoma (ILC), pre-operative detection of the lymph node metastasis may be demanding due to its unique morphology. The aim of this study was to examine the benefit of preoperative axillary ultrasound (AUS), ultrasound-guided fine-needle aspiration biopsy (US-FNAB), and intra-operative imprint cytology (IIC), in order to avoid two-stage axillary surgery in patients with ILC undergoing mastectomy.

**Methods:**

The object of this study were 102 patients (median age 52, range 34–73 years) with clinically non-suspicious axilla in whom 108 mastectomies were performed after a pre-operative AUS investigation. Whenever a metastasis was detected in a sentinel lymph node, ALND was done. Reports of the pre-operative AUS investigation, US-FNAB, and IIC were compared with definitive histopathological reports of surgical specimens.

**Results:**

In 46 cases lymph node metastases were diagnosed. AUS suspicious lymph nodes were found in 29/108 cases and histopathology confirmed metastases in 22/30 cases. US-FNAB was performed in 29 cases with AUS suspicious lymph nodes. Cytology proved metastases in 11/29 cases. Histopathology confirmed metastases in 10/11 cases with only isolated tumor cells found in one case. IIC investigation was performed in 63 cases and in 10/27 cases metastases were confirmed by histopathology. Pre-operative AUS, US-FNAB, and/or IIC investigation enabled ALND during a single surgical procedure in 20/46 patients with metastases in lymph nodes.

**Conclusion:**

Pre-operative AUS, US-FNAB, and/or IIC are/is beneficial in patients with ILC planned for mastectomy in order to decrease the number of two stage axillary procedures.

## Background

Breast cancer is a heterogeneous disease [1]. Invasive ductal carcinoma (IDC) is the most prevalent histological subtype of invasive breast cancer with invasive lobular carcinoma (ILC) following in the second place [2]. ILC is diagnosed in 5–15% of women with breast cancer [3]. Axillary lymph node status is a significant prognostic factor in breast cancer and has an influence on surgical and potential adjuvant treatment [4–6].

Axillary lymph node dissection (ALND) used to be part of surgical treatment in all breast cancer patients regardless of their axillary lymph node status [7]. ALND is associated with considerable postoperative complications and chronic morbidities including seroma, infection, lymphedema, sensory deficit, and loss of shoulder mobility [8–10]. Sentinel lymph node (SLN) biopsy is associated with less morbidity and has become the standard of care for patients with clinically negative axilla [7, 8]. Until the results of the ACOSOG Z0011 trial, patients with positive SLN were recommended to receive a completion ALND (cALND). With the introduction of Z0011 trial results, omission of ALND is safely considered in selected patients [7]. However, in patients undergoing mastectomy, positive SLN leads to ALND [8]. Pre-operative staging of the axilla is helpful in surgical planning and the treatment of these patients in hope of reducing the number of surgical procedures [11–17].

The aim of this study was to examine the benefit of pre-operative axillary ultrasound (AUS), ultrasound-guided fine needle biopsy (US-FNAB), and intraoperative sentinel node imprint cytology (IIC), in order to avoid two-stage axillary surgery in patients with ILC undergoing mastectomy.

## Methods

### Patients

**Fig. 1:**
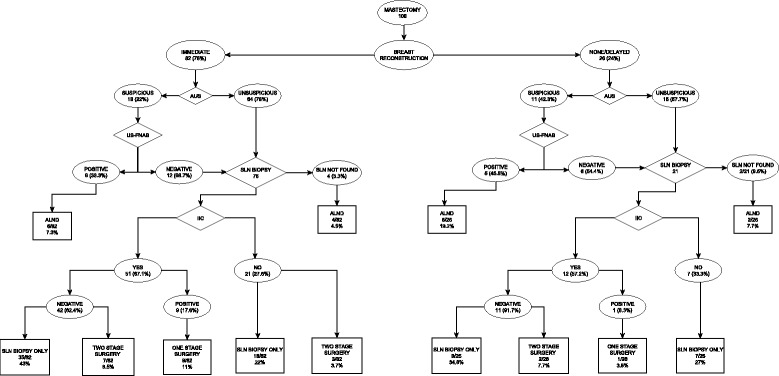
Flow chart of diagnostic and surgical procedures performed in the study population

A retrospective chart review of breast cancer patients treated at the Institute of Oncology Ljubljana from 2002 to 2015 was conducted. Patients from the National Breast Cancer Screening Program and the breast reconstruction database were included in the study. The object of this study were 102 women (median age 52, range 34–73 years) with pure ILC in whom 108 mastectomies were performed after a pre-operative AUS investigation. The clinicopathologic features of the study population and diagnostic and surgical procedures performed are described in Table 1 and Flow chart Fig. 1, respectively.

**Table 1:** Clinicopathologic features of the study population.

Clinical features	
Age (y)	
Median ± SD (range)	52 ± 8.7 (34–73)
< 50	42 (39%)
≥ 50	66 (61%)
Palpability of breast lesion	
Palpable	70 (64.8%)
Non-palpable	38 (35.2%)
Palpability of axillary lymph nodes	
Palpable	9 (8.3)
Non-palpable	99 (91.7%)
Multicentic breast lesion	
No	47 (43.5%)
Yes	61 (56.5%)
Pathologic features	
Median ± SD (range) [mm]	21.5 ± 17.1 (2–90)
T stage	
T1 (≤20 mm)	46 (42.6%)
T2 (> 20 mm, ≤50 mm)	46 (42.6%)
T3 (> 50 mm)	13 (12%)
T4	3 (2.8%)
N stage	
N0	62 (57.4%)
N1	27 (25%)
vN2	8 (7.4%)
N3	11 (10.2%)
Variant of ILC	
Classic	97 (90%)
Pleomorphic	11 (10%)
Histologic grade	
I	7 (6.5%)
II	88 (81.5%)
III	13 (12%)

### AUS and US-FNAB

Pre-operative AUS has been performed in all patients regardless of the clinical status of the axilla, and was performed as described in detail elsewhere [17]. Pre-operative AUS was performed by an experienced breast radiologist using a 12–15 MHz linear-array transducer. Recognised as suspicious were lymph nodes (LN) with longitudinal-transverse axis ratio < 1.5, cortical thickness of < 3 mm and/or where the hilus was not seen by AUS.

Whenever a suspicious LN was detected by AUS, a radiologist performed an US-FNAB with a 21-G needle and two smears were prepared. This was done in 29 cases. Traditionally, surgeons relied on results of FNAB and cytology at our Institute since 1970-ies and only definitive samples obtained by surgical procedure were examined by pathologists. So, for patients included in our study core biopsies of lymph nodes or frozen section of sentinel lymph nodes have not been done.

### Patient treatment

Neoadjuvant chemotherapy was administrated in only two patients at the discretion of the medical oncologist. All patients underwent mastectomy and majority had immediate or delayed breast reconstruction.

SLN biopsy was performed in patients with negative cytology on US-FNAB and/or unsuspicious AUS report. A standard double indicator technique was used to identify the SLN. On the morning of surgery, 30–60 MBq of ^99m^Tc labelled nanocolloid (Nanocoll) in 0.2 ml saline, divided in two doses, was injected peritumorally at two sites [18], while 1 ml of Patent blue (Blue Patente V; Laboratorie Guerbet, Aulnaysous-Bois, France) was injected peritumorally only few minutes prior to the surgery.

IIC was performed at the discretion of the surgeon. It was performed in patients with a palpable and/or large non-palpable breast lesion. IIC was not done in patients with a small non-palpable ILC. For the intraoperative examination of SLN the touch imprint cytology was used. Excised sentinel LNs were sent to the pathology department to be intraoperatively examined. Each sentinel LN was bisected along the long axis and then sectioned transversely at 2 mm. From each slice, imprints were made by gently touching the cut surface of sentinel LN to a glass slide. The imprints were air-dried and stained with quick stain (Hemacolor, Merck KgaA, Darmstadt, Germany). A board-certified cytopathologist examined the imprints and diagnosed them as negative, suspicious, or positive.

ALND was performed if a cytopathologist confirmed malignant cells in a lymph node sample obtained by US-FNAB or IIC. Moreover, ALND was also performed if sentinel LN was not successfully identified by lymphoscintigraphy. According to the TNM staging system [19], a nodal metastasis was defined as a macrometastases (> 2.0 mm) or a micrometastases (> 0.2 mm and ≤2.0 mm). Lymph nodes with isolated tumour cells (ITC) (≤0.2 mm) were regarded as negative. Reports of pre-operative AUS investigation, US-FNAB and IIC were compared with a definitive histopathological report of the surgical specimens. The results are reported on a per patient basis.

### Statistical analysis

The association between categorical variables was tested by the Pearson chi-square test or Fisher’s exact test, as appropriate. All comparisons were two-sided and the *p*-value < 0.05 was considered statistically significant. Sensitivity, specificity, positive predictive value (PPV), negative predictive value (NPV), false negative rate (FNR) and false positive rate (FPR) were calculated to evaluate the ability of AUS, US-FNAB, and IIC to detect lymph node metastases. Statistical analyses were performed using the SPSS software (IBM Corp., version 22.0 Armonk, NY).

## Results

A total of 102 women with pure ILC in whom 108 mastectomies were performed and who had undergone pre-operative AUS were identified. The majority of patients were older than 50 years (61%). Most patients had palpable breast tumour (65%) with clinically negative axilla (92%). In majority of patients oestrogen and progesterone receptors were positive (99% and 92%, respectively).

A breast reconstruction was performed in 88 patients with 82 patients undergoing immediate reconstruction. Immediate reconstruction with implants was performed in 33/82 patients and immediate autologous reconstruction in 49/82 patients. In patients with immediate breast reconstruction an immediate ALND was done in 19 cases and delayed cALND was done in 10 cases. An immediate axillary lymphadenectomy was done whenever feasible through the same incision, while a delayed cALND was always done through a separate axillary incision.

On final pathology tumour diameter was ≥11 mm in 91% and ≥21 mm in 52%. From 1 to 6 (median 1, mean 2) sentinel LN were harvested. Overall, in 46/108 cases (43%) LN metastases were confirmed. Micrometastases represented 26% and macrometastases 74% of all identified metastasis on final pathology. Node positive disease was found in 32% of patients with palpable and 10% of patients with non-palpable breast lesion (*p* = 0.066). Moreover, when breast lesion was larger than 20 mm, nodal disease was found in 30% of patients (*p* = 0.001).

Using AUS, suspicious LNs were found in 28% of included patients and in 20% nodal disease was confirmed on the final pathology (*p* = 0.0001).

US-FNAB investigation was performed in 29 cases. It was positive in 11 cases and ALND was performed due to the positive cytology. Histopathology confirmed metastases in 10/11 cases, while in one case ITC were found. When LNs were suspicious on AUS, but cytology was negative, histopathology confirmed metastases in 6 cases where reactive lymphadenitis was reported by cytology and in 4 cases with inconclusive cytology due to the non-diagnostic US-FNAB material. Accuracy and sensitivity of US-FNAB significantly changed with the extent of the nodal disease and were 59% and 25% for N1 disease compared to 88% and 87.5% for N3 disease, respectively (*p* = 0.008).

The results of AUS and US-FNAB detection of nodal metastasis relative to tumour stage are described in detail in Table 2. Overall sensitivity, specificity, PPV, NPV, FNR and FPR of AUS and US-FNAB were 49%, 87%, 73%, 70%, 0.59, 3.85 and 50%, 89%, 91%, 44%, 0.56, 4.5, respectively. The sensitivity of AUS and US-FNAB in regard to the size of nodal metastasis is described in Table 3. In general, US-FNAB enabled single axillary procedure in 7, 1 and 2 patients with N3, N2 and N1 disease, respectively.

**Table 2:** The results of AUS and US-FNAB detection of nodal metastasis relative to tumor stage

Tumor stage	pN+	No. of positive axillas detected by AUS	Sensitivity (%)	Specificity (%)	p-value	pN+	No. of positive axillas detected by US-FNAB	Sensitivity (%)	Specificity (%)	p-value
T1	12	4	33	97	0.0001	3	2	67	100	0.116
T2	22	9	41	83		9	2	22	100	
T3	10	8	80	67		7	5	71	100	
T4	1	1	100	0		1	1	100	50

**Table 3:** The sensitivity of AUS and US-FNAB for detection of nodal metastasis relative to the size of lymph node metastasis

Size of lymph node metastasis	Cases with pN+	AUS suspicious	AUS sensitivity (%)	p-value	US-FNAB positive	US-FNAB sensitivity (%)	p-value
≤0.2 mm	20	5	25	0.000	1	20	0.002
> 0.2 mm and ≤2.0 mm	12	3	25		0	0	
> 2.0 mm without extracapsular extension	14	6	43		3	33	
> 2.0 mm with extracapsular extension	20	12	60		7	58	

IIC investigation was performed in 63 cases. It was positive in 10/27 cases with metastasis diagnosed on final pathology. Tumor characteristics in patients, where IIC was performed and the results of IIC detection of nodal metastasis relative to tumor stage and relative to different variants of ILC are described in Table 4. The overall sensitivity, specificity, PPV, NPV and FNR of IIC were 37%, 100%, 100%, 68% and 0.63, respectively. IIC was positive in 1/11 (9%) patients with micrometastasis, in 4/7 (57%) with macrometastasis without extracapsular invasion and in 5/9 (56%) patients.with macrometastasis and extracapsular extension. Due to the IIC, a two stage axillary procedure was avoided in 8 and 2 patients with N1 and N2 disease, respectively.

**Table 4:** Tumor characteristic and the results of intraoperative imprint cytology detection of nodal metastasis

	IIC positive	IIC negative	Cases with pN+	Cases with pN-	p-value
Underwent IIC	10	53	27	36	0.0001
Age (years)					
< 50	5	22	11	16	0.733
≥ 50	5	31	16	20	
Palpability of breast tumor Palpable	9	38	23	24	0.429
Non-palpable	1	15	4	12	
Multicentic breast lesion					
No	2	24	10	16	0.175
Yes	8	29	17	20	
Size of lesion at surgery					
≤ 20 mm	2	26	7	21	0.164
> 20 mm	8	27	20	15	
T stage					
T1	1	24	6	19	0.085
T2	7	25	17	15	
T3	2	4	4	2	
Variant of ILC					
Classic	10	48	26	32	0.583
Pleomorphic	0	5	1	4	

Among 63 cases with IIC immediate ALND and another surgical procedure for cALND was done in 10 (16%) and 9 (14%) cases, respectively. On the other hand, among 28 cases with SLN biopsy but without IIC none cases had immediate ALND, while 3 (11%) cases had another surgical procedure for cALND.

Charges for procedures in a cost-analysis were taken into account as follows: AUS investigation 60 EUR, US-FNAB 140 EUR, IIC 200 EUR and cALND 2500 EUR. Cost to perform AUS/US-FNAB/IIC in 100% of patients would be smaller (43.180 EUR) in comparison to total cost associated with avoiding a second operation in 43% of patients (115.000 EUR).

## Discussion

Pre-operative staging of the axilla is useful in surgical planning and the treatment of patients undergoing mastectomy in hope of avoiding completion ALND. However, studies in patients with ILC evaluating the diagnostic performance of AUS, US-FNAB, and/or IIC are limited [11–16, 20–22]. In this study, the value of pre-operative AUS, US-FNAB, and IIC was examined in patients with ILC undergoing mastectomy, in order to avoid two-stage axillary surgery. The study population was comparable with other similar published studies in the number of included patients with ILC, their age, the size of primary breast lesion, and the number of patients with node involvement on final pathology [13–16]. In this study the overall rate of identifying patients with ILC who would require ALND was 43% using before mentioned pre-operative and intraoperative diagnostic modalities.

In our study, the overall sensitivity of AUS in detecting LN metastases was 49%. The results are comparable with the results of other similar studies where the reported sensitivity of AUS in detecting lymph node metastasis ranged from 36% to 68% [11, 13–16] Moreover, the reported sensitivity of US-FNAB in detecting lymph node metastases differed even more in published studies, ranging from 29% to 78% [12–14, 16]. In this study, the sensitivity of US-FNAB was 50%. One should expect that with core biopsy and histological sample obtained from suspicious LN, detection of the ILC within the LN is easier. Even when core biopsy was used, accurate pre-operative identification of LN metastases was made in 48% in patients with ILC [15], which is similar to our study. But authors report that even when core biopsy was used instead of US-FNAB, the sensitivity for detecting ILC node metastasis was similar to sensitivity of US-FNAB (33%) [11]. Moreover, core biopsy, being more invasive method, can result in higher complication rate in the unexperienced hands. Therefore, changing the method for the pre-operative LN sampling in the ILC patients from US-FNAB to core biopsy in hope of improving the sensitivity of the pre-operative axillary staging, must be taken with care. At our institution a core biopsy of axillary lymph nodes or intraoperative frozen section of SLN was not done at all because during that period according to our national guidelines for breast cancer, FNAB or US-FNAB were the standard of care for all breast cancer patients with palpable and/or US suspicious axillary lymph nodes. These two modalities represented the standard of care for breast cancer patients in our country. However, in light of ACOSOG Z011 trial there is less need for IIC and cytologists will in future generate less experience with IIC.

A rising success rate of AUS and US-FNAB detecting nodal ILC metastases is observed in patients with larger tumors. In our study, the sensitivity of AUS significantly improved from 38% for T1/T2 staged tumors to 82% for T3/T4 staged tumors (*p* = 0.0001; Table 2). The improvement corresponds with the observed improvement in AUS sensitivity described in Boughey’s study (from 47% to 55%) [14]. Our data shows that the sensitivity of US-FNAB for T1/T2 tumors and T3/T4 tumors was 33% and 75%, respectively. The improvement in US-FNAB sensitivity for larger T-stages is in agreement with the results observed in other studies (87% to 97% and 47% to 64%, respectively) [14, 16]. Moreover, in our study, a statistically significant improvement of US-FNAB sensitivity was also observed in N2/3 disease (67%) compared to N1 disease (25%). A similar improvement of sensitivity was described by Topps et al. where an improved sensitivity was observed in N2/N3 disease (59%) compared to N1 disease (45%) [16]. Cost-analysis in our cohort of patients showed that our treatment algorithm was cost effective in comparison to cALND done as separate surgical procedure. Based on our data and data from the literature it is obvious that in patients with more advanced disease AUC, US-FNAB and IIC are more cost effective.

Furthermore, the size of LN metastases was correlated with AUS and US-FNAB detection rate for correctly identifying nodal metastases. An improvement of sensitivity was observed as morphological changes within metastatic LN became more prominent. In this study, the sensitivity of AUS significantly improved with the size of nodal metastasis and was 25% for micrometastases, 43% for macrometastases without extracapsular extension, and 60% for macrometastases with extracapsular extension (*p* = 0.0001). Furthermore, the sensitivity of US-FNAB was 0%, 33% and 58% for micrometastases, macrometastases without extracapsular extension, and macrometastases with extracapsular extension (*p* = 0.002), respectively. In comparison, Boughey also reported a higher sensitivity with a lower false negative rate of AUS and US-FNAB in patients with larger nodal metastasis [14]. The sensitivity of AUS and US-FNAB was 13% for micrometastases and 48% for macrometastases larger than 5 mm [14].

Different techniques are used to examine the SLN intraoperatively. Some authors advocate IIC for detection of metastasis in SLN in patients with ILC undergoing mastectomy that have otherwise negative AUS and negative US-FNAB [20–22]. With IIC SLN, metastases can be rapidly and reliably detected during operation and ALND can be performed in a single operation [18, 20]. In reported series, the number of patients with ILC was small and comparable to our study [20–22]. IIC had sensitivity from 52% to 71% for intraoperative detection of metastases in SLN [20–22]. However, the sensitivity of IIC in our study was only 37%. It corresponds with the reported sensitivity of 34% published in another, larger study performed at our Institute, which evaluated the use of IIC in patients with majority IDC breast cancer patients with IDC and ILC [18]. Low sensitivity of IIC in our Institute was probably due to the different preparation and fixation technique used for IIC at our Institute. In our study, the sentinel LN fat capsule was not always completely removed as described in other studies [21]. Furthermore, all our imprints were air-dried and stained with Hemacolor quick stain. Other studies with a higher sensitivity of IIC reported the use of two techniques combined, one being air drying and quick staining and the other an immediate 3-min fixation with ethanol and staining with hematoxylin and eosin (HE) [20, 22], or only using 3-min ethanol fixation with HE staining [21].

Another technique for detection of metastases in SNB is frozen section of SNB [23]. Horvath et al. reported that frozen section has sensitivity of 70% for detection of metastases in SNB in patients with ILC, so they conclude that frozen section analysis of breast cancer patients should remain the standard of care [23]. However, Howard-McNatt et al. published even higher sensitivity (71%) using IIC for detection of metastases in SNB in a series of patients with ILC [22].

In our study, IIC sensitivity significantly improved with larger metastases within the lymph node and was 9% for multiple micrometastasis and 57% and 56% for macrometastasis without and with extracapsular extension, respectively (*p* = 0.0001). Our results are in agreement with the results of Creager’s study where the sensitivity of IIC improved with the size of nodal metastasis and was 33% for micrometastasis and 73% for macrometastasis [20]. Moreover, in our study, the sensitivity of IIC also improved with tumor stage from 17% for T1 staged tumors to 50% for T3 staged tumors; the difference was close to being statistically significant (*p* = 0.087). On the other hand, in Creager’s study, no statistical difference was found between the accuracy of IIC detection of nodal metastasis in regard to tumor stage (*p* = 0.82) [20]. The difference in the statistical significance is probably due to the fact that patients with larger tumors were included in our study. The mean tumor diameter in our patients was 26 mm, while in Creager’s patients it was only 18 mm [20]. In our series IIC was not done in all patients. It was performed in patients with a palpable and/or large non-palpable breast lesion. IIC was not done in patients with a small non-palpable ILC. It might potentially act as a confounding factor (selection bias) for lower detection rate of CII in smaller tumors in our series in comparison to Creager’s series. However, our data suggest that the preoperative and intraoperative diagnostic approach should be done also in patients with small ILC. Another argument for this approach is cost-effective analysis which showed that our diagnostic algorithm has 2.66 times lower price in comparison to cALND done as a separate surgical procedure.

Furthermore, Creager et al. [20] also investigated the correlation between the accuracy of nodal metastasis detection by IIC and different ILC variants. The difference in sensitivity of IIC was reported for classic versus pleomorphic ILC (47% and 75% respectively) [20]. In our study, the number of patients with classic and pleomorphic ILC were similar to Creager’s, the results being completely opposite, with the sensitivity of IIC for classic ILC of 38% and pleomorphic ILC of 0%. Like in Creager’s study, the number of included patients in these groups was too small for reaching statistical significance.

Our study has limitations. First of all, due to the retrospective design of the study, AUS protocols were not the same for all included patients and more than one radiologist has been involved with pre-operative axillary staging of included patients. Moreover, the number of patients with nodal involvement was low, with N2/N3 disease present in only 18% of included patients, which affected the reported sensitivity of AUS, US-FNAB and IIC. Ideally, the most meaningful comparison of our results for IIC would be to compare it with a separate cohort who was not a part of the treatment algorithm at our Institute. Unfortunately, at our Institute we don’t have experience with intraoperative SLN frozen section and there is no separate cohort. During the study period all our patients followed the same treatment protocol as was recommended by our national guidelines for breast cancer patients. Therefore our results were compared only with figures from the literature.

The detection of axillary LN metastasis in patients with ILC may be further improved with the use of new sonographic techniques [24–26]. Elastography and contrast-enhanced ultrasound (CEUS) could be emerging sonographic modalities that would allow the detection of metastatic axillary lymph nodes with preserved architectural structure but changed elasticity and vascular pattern of the infiltrated metastatic nodal tissue and therefore detection of N1 disease [24–28]. Furthermore, the preparation and fixation techniques for IIC in our study greatly differed from the techniques reported by other authors. Improved sensitivity of IIC could be obtained with the implementation of better fixation techniques reported by other authors. For these purposes, a prospective study including new ultrasound modalities with the implementation of improved IIC techniques should be conducted for the evaluation of pre-operative and intraoperative staging of the axilla in ILC patients.

One can argue that in the light of changing protocols for the treatment of axilla in breast cancer patients, where ALND is replaced by radiotherapy in selected patients, pre-operative and intraoperative detection of LN metastasis may be redundant. However, it must be emphasized that in our study majority of patients, who benefited from one stage axillary procedure were patients with N2/N3 disease and patients with large LN metastases, who will, despite the changes in the axillary treatment, still require ALND as part of the regional treatment of the nodal disease.

## Conclusion

Pre-operative confirmation of nodal metastasis in a patient undergoing mastectomy enables immediate ALND and spares a two-stage surgery. The detection of axillary lymph node metastasis in patients with ILC is demanding due to its unique morphologic features. Pre-operative identification of axillary lymph node metastasis is challenging and often unsuccessful despite using pre-operative axillary staging with AUS and US-FNAB. IIC using air-drying and staining with Hemacolor quick stains in ILC patients probably has a limited value. AUS, US-FNAB, and IIC should be carried out in patients with ILC undergoing mastectomy, because they enabled a one-stage surgical procedure in 20/46 patients with metastases in lymph nodes.
